# New COVID-19 vaccination recommendations in Spain: Optimizing for next seasons

**DOI:** 10.1016/j.eimc.2024.08.012

**Published:** 2025-01

**Authors:** Pilar Arrazola, María Fernández Prada, Ángel Gil, José Gómez Rial, Cristina Hernán, Rosario Menéndez, Antoni Trilla, Raúl Ortiz de Lejarazu

**Affiliations:** a Servicio de Medicina Preventiva, Hospital Universitario 12 de Octubre, Madrid, Spain; b Servicio Medicina Preventiva y Salud Pública, Hospital Vital Álvarez Buylla, Asturias, Spain; c Departamento de Especialidades Médicas y Salud Pública, Universidad Rey Juan Carlos, Madrid, Spain; d Servicio de Inmunología, Hospital Clínico Universitario de Santiago de Compostela, Servicio Galego de Saúde (SERGAS), Galicia, Spain; e Servicio de Medicina Preventiva y Salud Pública, Hospital Clínico Universitario de Valladolid, Valladolid, Spain; f Instituto de Investigación La Fe, Valencia, Spain; g Hospital Clínic, Universitat de Barcelona, ISGlobal, Barcelona, Spain; h Servicio de Medicina Preventiva y Epidemiología, Hospital Clínic, Universitat de Barcelona, Spain; i Centro Nacional de Gripe de Valladolid (GISRS/WHO), Spain

**Keywords:** COVID-19, SARS-CoV-2, Omicron, Vaccination, Recommendations, Spain, COVID-19, SARS-CoV-2, Ómicron, Vacunación, Recomendaciones, España

## Abstract

Despite high initial vaccination rates, Spain's current COVID-19 vaccination coverage in recommended groups does not meet WHO targets. For the upcoming season, challenges include revising vaccination age, updating risk groups, and unifying criteria with flu vaccine co-administration. European Commission's advance purchase agreements limit access to certain vaccines, and the need for vaccines effective against current variants adds administrative complexities. Spain's COVID-19 vaccination recommendations should adapt to these specific circumstances. Using vaccines effective against predominant variants with appropriate response duration is crucial to protect at-risk populations. Enhancing training and health education campaigns for health professionals and the general public, alongside utilizing tools to simplify vaccination recommendations, can promote higher vaccination rates in Spain. Addressing these challenges is essential to ensure adequate protection and improve vaccination coverage, ultimately achieving better public health outcomes in the face of evolving COVID-19 threats.

## Abstract (es)

A pesar de las altas tasas de vacunación alcanzadas durante los primeros años de pandemia, actualmente en España la cobertura de vacunación contra la COVID-19 en los grupos recomendados no alcanza los objetivos propuestos por la Organización Mundial de la Salud (OMS). Algunas de las dificultades que deben abordarse para la temporada de vacunación de 2024-2025 son la edad de vacunación, la revisión de los grupos de riesgo y la unificación de criterios con la vacuna de la gripe para su administración conjunta. Además, los acuerdos de compra anticipada establecidos por la Comisión Europea que limitan el acceso a ciertas vacunas y la necesidad de vacunas actualizadas a las variantes que circulan hoy en día, añaden otras dificultades de orden administrativo para la temporada de vacunación de 2024-2025. Las recomendaciones para la vacunación de la COVID-19 en España deben adaptarse a estas circunstancias específicas y emplear vacunas eficaces contra las variantes predominantes con una duración de la respuesta lo más prolongada posible para maximizar la protección de la población de riesgo. La formación y las campañas de educación sanitaria para profesionales sanitarios y población general, además de emplear herramientas para simplificar las recomendaciones pueden promover una mayor tasa de vacunación en la población española.

## Introduction

The COVID-19 pandemic generated important changes in daily life, work and society globally. Since its emergence, this virus has had a significant impact on public health, the economy and society. As the scientific and medical community continues to investigate this disease, the complexity of COVID-19 and its long-term consequences are being recognized.

Severe forms of COVID-19 have resulted in a high number of hospitalizations, intensive care unit (ICU) admissions, and deaths throughout the pandemic. From March 28, 2022, to July 5, 2023, 162,409 hospitalized COVID-19 cases, 6763 ICU admissions, and 17,902 deaths were reported in Spain.^1^ Although the pandemic has ended in its acute phase, the risk of infection remains; a recent study in the United States confirms that in the 2023–2024 season, the risk of death in hospitalized COVID-19 patients was higher than in those hospitalized for influenza.^2^

At present, although its incidence and mortality have declined significantly, severe forms continue to be concentrated in certain at-risk populations (over 60 years of age, severe chronic pathologies and immunosuppression) with unacceptably high rates of mortality surpassing influenza. Moreover, COVID-19 has demonstrated the ability to cause prolonged and debilitating symptoms in a significant number of patients, a condition that has been termed “persistent COVID”, post-acute COVID syndrome (PACS) or “long COVID”. This complication occurs in at least 10% of patients,^3^ and poses additional challenges for both sufferers and healthcare systems, as patients may experience a wide range of persistent symptoms including fatigue, respiratory difficulties, cognitive problems and neurological disorders, among others.^3^

In this context, vaccines emerged as a crucial tool in the fight against the pandemic, making it possible to reduce the spread of the virus and especially the disease burden. Vaccines against COVID-19 have been successfully shown to reduce infection rates, severity, hospitalization and mortality among different populations by 80 to >90%.^4^ While licensed vaccines have been shown to be highly effective, their ability to prevent prolonged COVID is not well understood. However, evidence suggests that vaccination helps mitigate the long-term effects of the virus,^5^ although more research is needed to confirm this hypothesis, and of course, each SARS-CoV-2-prevented infection or illness eliminates the possibility of persistent COVID.^6^

In Spain, one of the countries most affected by the pandemic in Europe^7^ vaccination coverage is decreasing due to a false sense of the disappearance of the virus, a lack of perception of the seriousness of the disease, and a decrease in the strength of the recommendation of vaccination by health professionals. All this contributes to a cessation of preventative measures leaving the at-risk population unprotected.

This article will discuss some of the current challenges in vaccination against COVID-19, the obstacles in the interpretation and implementation of current vaccination recommendations, the types of vaccines available, and how to increase, through different actions, vaccination coverage in Spain.

## Epidemiology and data on COVID-19 vaccination in Spain

Although June 2023 data from the Spanish Health Alerts and Emergencies Coordination Center show a downward trend in the incidence of COVID-19 in Spain, transmission of the virus persists at moderate levels, with significant increases in cases associated with different variants of the Omicron lineage and its derived variants such as JN.1. Despite a decrease in hospital and ICUs occupancy, it remains high in some regions. SARS-CoV-2 is still far from being stationary and no one can anticipate future epidemic upsurges.^8^

In the most vulnerable populations, the elderly, people with comorbidities or immunocompromised, there is still a high risk of serious complications, hospitalization or death from COVID-19, highlighting the need for specific protection strategies and equitable access to vaccination for these high-risk groups.^8^

The recommended targets for influenza and COVID-19 vaccination in the 2023–2024 season were to achieve or exceed vaccination coverage of 75% in persons over 60 years of age and in healthcare professional, as well as to exceed 60% in pregnant women and persons at risk.^9^ Despite this, in January 2024, the percentage of people over 60 years of age with up-to-date vaccine doses in Spanish territories was 46%, and only reached 64.3% in people over 80 years of age.^10^ In the 2023–2024 season, vaccination coverage among healthcare professionals stood at 13.9%.^11^

In Spain, a higher number of influenza vaccines administered has been observed in comparison with COVID-19. Data from some autonomous communities, such as Catalonia and Andalusia, show this difference: in Catalonia in the 2023–2024 season, 80,000 more people over 60 years of age were vaccinated for influenza than for COVID-19, and in Andalusia this difference was more than 150,000 for this age group.^12^, ^13^

In this context, it is important to adapt control and prevention strategies to address the needs of these vulnerable populations. This includes the design of targeted vaccination programs, as well as the provision of adequate resources and support for their proper implementation.^14^

## Current recommendations in Spain and Europe (2024–2025)

### Recommendations in Spain

**Fig. 1:**
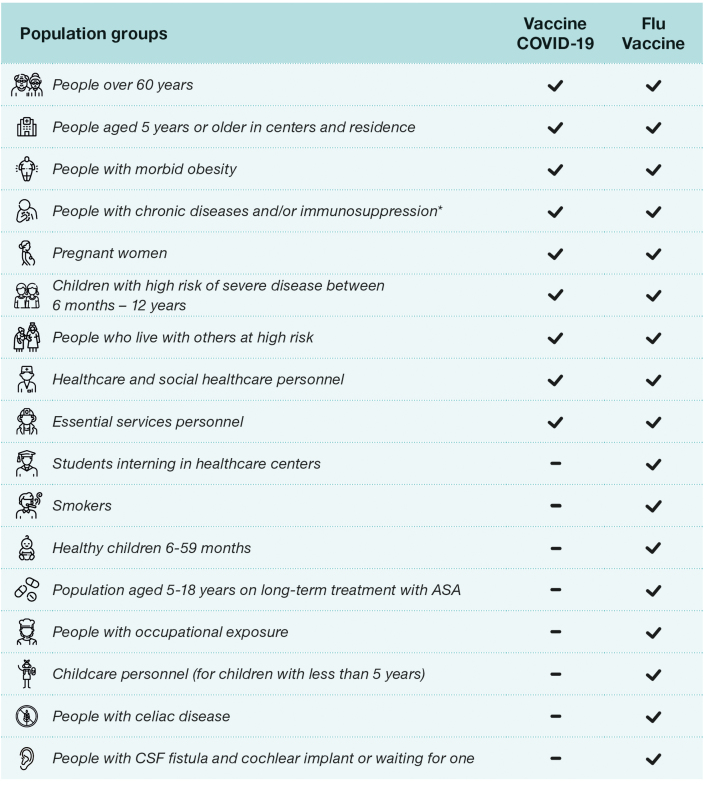
COVID-19 and influenza vaccination recommendations season 2024–2025. ASA: acetylsalicylic acid, CSF: cerebrospinal fluid. *Upon 12 years, including diabetes mellitus and Cushing's syndrome; morbid obesity (body mass index ≥ 40 in adults, ≥35 in adolescents or ≥3 SD in childhood); chronic cardiovascular (not excluding hypertension) neurological or respiratory chronic diseases, including: bronchopulmonary dysplasia, cystic fibrosis, and asthma; chronic kidney disease and nephrotic syndrome; hemoglobinopathies and anemias or hemophilia, other coagulation disorders and chronic bleeding disorders, as well as recipients of blood products and multiple transfusions; asplenia or severe splenic dysfunction; chronic liver disease, including chronic alcoholism; severe neuromuscular diseases; immunosuppression (including primary immunodeficiencies and that caused by HIV infection or drugs, as well as in transplant recipients and complement deficiency); cancer and hematological malignancies; cerebrospinal fluid fistula and cochlear implant or awaiting implant; celiac disease; chronic inflammatory disease; disorders and diseases leading to cognitive dysfunction: Down's syndrome; dementias and others.

The recent Spanish recommendations for vaccination against COVID-19 approved on July 18, 2024, are carried out in conjunction with influenza vaccination, as well as last year's recommendations.^9^, ^15^ The recommendations are broader for influenza vaccination than for COVID-19 vaccination, which makes co-administration difficult as they have different requirements. In the case of influenza vaccine (Fig. 1), it also includes a recommendation for all children between 6 and 59 months of age, and those between 5 and 18 years of age with chronic treatment with acetylsalicylic acid, smokers, students in health and social-health centers, people with direct occupational exposure to animals or their secretions and nursery and kindergarten staff (under 5 years of age).^9^ Something that has changed in the current guidelines with respect to the previous year is that in patients with cerebrospinal fluid fistula and cochlear implant or awaiting implant, and Celiac disease is currently recommended vaccination only against influenza, and COVID-19 vaccination has been excluded.^15^

An important point that needs to be clarified by the Spanish health authorities is which criteria define the need for an additional dose in immunosuppressed patients, as the current recommendations are open to interpretation by the clinician.^15^

It should be noted that, except in particular cases, the recommendations issued for the 2024–2025 campaign called for the use of updated monovalent vaccines with Omicron JN.1 variant as antigen and mRNA and recombinant protein vaccines will be available. Something new of the current recommendations is that recombinant protein vaccine will be administered preferably in the population aged 80 years and older and institutionalized in nursing homes.^15^

In this regard, the World Health Organization (WHO) and the European Medicines Agency (EMA) also have recently recommended the use of monovalent vaccines adapted to the JN.1 variant and its lineages for the 2024–2025 season, given the global predominance of this variant in the first months of 2024.^16^, ^17^

### Recommendations in other European countries

In Table 1 the recommendations issued by Spain and different European countries regarding vaccination against Influenza and COVID19 can be compared. In general, vaccination against COVID-19 is recommended in patients over 60 years of age, residents in long-term care centers, close contacts of patients at risk, healthcare professionals and patients with immunosuppression. It is important to note that in some countries an additional dose is recommended in the spring for patients at increased risk of severe disease.^15^, ^18^, ^19^, ^20^, ^21^, ^22^, ^23^

**Table 1:** COVID-19 vaccination recommendations in some European countries.

Country	Authority	Timing	Population(s)	Products and/or composition	Coadministration flu vaccines
France^18^	Sanitary authority (Haute autorité de santé)	**Spring** (annual routine) Publication: 26th April 2024	• Adults aged 80 years and older • Immunocompromised patients of any age • Residents of homes for the dependent elderly and long-term care unit, regardless of age	• Comirnaty XBB.1.5 • Nuvaxovid XBB.1.5	Yes ✓
		**Autumn** (annual routine) Publication: 26th April 2024	• Routine, annual autumn COVID-19 vaccination for all adults aged 65+ years and older • At risk of severe disease, including: ̊ Persons aged 6+ months and older with co-morbidities that put that at higher risk for severe outcomes (e.g., hypertension, heart, liver, lung and kidney diseases, cancer, diabetes, etc.) ̊ Immunocompromised persons ̊ Pregnant women ̊ Residents of long-term care facilities and nursing homes ̊ Close contacts of immunocompromised or at-risk persons	• Comirnaty XBB.1.5 • Nuvaxovid XBB.1.5	Yes ✓

Germany^19^	Standing Committee on Vaccination (STIKO)	**Autumn 2023** (annually) Publication: 25th May 2023	• Adults aged 60 years and older • Residents in care facilities and people with an increased risk of stroke • People aged 6 months and older with an underlying disease • Persons of any aged with an increased work-related risk of infection in medical and/or nursing care with direct contact to patients or residents • Family members and close contacts from the age of 6 months of persons who are not expected to have a protective immune response after a COVID-19 vaccination	• mRNA or protein-based • With a variant adjustment currently recommended by the WHO	Yes ✓

Italy^20^	Ministry of Health	**Autumn 2023** Publication: 14th August 2023	• Adults aged 60 years and older • Residents of long-term care facilities • Pregnant and lactating women • Health and social care workers working in hospitals, community and long-term care facilities; medical students • People aged 6 months to 59 years with high frailty as a result of diseases or conditions that increase the risk of severe COVID-19	• mRNA XBB.1.5 • Protein-based XBB.1.5	Yes ✓
		**Autumn 2024**	Not yet published	NA	NA

Spain^9^, ^15^	Public Health Commission	**Autumn 2023** Publication: 12th September 2023	• Adults aged 60 years and older • Persons aged 5 years and over in disability centers and old peoples’ homes • Persons aged less than 60 years with specific risk conditions • Pregnant and postpartum women • Persons with a high degree of immunosuppression • Health and social care workers working in hospitals	• Newly adapted vaccines (e.g., XBB mRNA or protein-based)	Yes ✓
		**Autumn 2024–2025(annual)**: 18th July 2024	• Adults aged 60 years and older • Persons aged 5 years and over in disability centers and old peoples’ homes • Persons 12 years of age and older with specific risk conditions • Pregnant and postpartum women • Persons with a high degree of immunosuppression • Health and social care workers working in hospitals • Children between 6 months and 12 years of age with conditions associated with an increased risk of severe disease and family members living with them	Newly adapted vaccines (e.g., JN.1-lineage mRNA or protein-based^a^)	Yes

United Kingdom^21^, ^22^, ^23^	Joint Committee on Vaccination and Immunization (JCVI)	**Autumn 2023** Publication: 30th August 2023	• Residents in a care home for older adults • All adults aged 65 years and over • Persons aged 6 months to 64 years in a clinical risk group • Frontline health and social care workers • Persons aged 12–64 years who are household contacts of people with immunosuppression • Persons aged 16–64 years who are carers and staff working in care homes for older adults	• Spikevax BA.4-5 • Spikevax XBB.1.5 • Comirnaty BA.4-5 • Comirnaty XBB.1.5 • VidPrevtyn Beta	Yes ✓
		**Spring 2024** Publication: 7th February 2023	• Adults aged 75 years and over • Residents in a care home for older adults • Individuals aged 6 months and over who are immunosuppressed	• Comirnaty XBB.1.5 • Spikevax XBB.1.5 • Nuvaxovid (as second-line) • Bimervax (as second-line)	NA

NA: not applicable.

a Recommendations is that recombinant protein vaccine will be administered preferably in the population aged 80 years and older and institutionalized in nursing homes.

There are differences between the recommendations within European countries, such as in the inclusion or exclusion of pregnant and lactating women or medical students, both of which are recommended in Italy, but not in Spain.^15^, ^18^, ^19^, ^20^, ^21^, ^22^, ^23^

On the other hand, the European recommendations, including the Spanish ones, take into account the continuous evolution of SARS-CoV-2 and the appearance of new variants, of the Omicron XBB.1.5 lineage in the 2023–2024 season and JN.1 lineages in the upcoming 2024–2025 season.^16^, ^17^ Hence, the importance of having updated vaccines that can offer effective protection against the strains circulating at any given time.^15^, ^18^, ^19^, ^20^, ^21^, ^22^, ^23^ This updating of vaccine strains is essential to maintain the effectiveness of vaccination strategies in protecting target populations. In addition, different patient profiles, including high-risk groups and those with underlying medical conditions, require the availability of different types of vaccines that are tailored to the particular needs of each individual.^24^

## Need to diversify the vaccine portfolio in Europe

Once the acute phase of the pandemic is over, it is necessary to fine-tune as much as possible the different vaccine options against COVID-19 in order to optimize their overall effectiveness and enable improvements in the resulting immunity.

The ability to choose between different vaccine platforms allows for greater personalization of vaccination, thereby optimizing immune response and ensuring adequate protection for the entire population and also helps avoid supply-chain problems that can occur if reliance is placed on a single supplier. This adaptive, patient-centered approach is essential to address the evolving challenges of the pandemic and promote equity in access to vaccination.^25^

During the pandemic, the EU Council of Health Ministers agreed on the need for joint action to support the development and distribution of COVID-19 vaccines for European Member States.^26^ To implement this action, the Commission created “*advance purchase* *agreements*” (APAs) for COVID-19 vaccines with several vaccine manufacturers. The intention was to maintain this agreement for the duration of the pandemic; however, this agreement was amended in May 2023 between the European Commission and Pfizer/BioNTech-Pfizer to alleviate purchasing obligations and resolve the ongoing dispute over the oversupply of COVID-19 vaccines.^27^ Although the details of the revised terms of the APA were not publicly disclosed, it is inferred from the terms of the contract that approximately 70 million doses of Comirnaty® (BioNTech/Pfizer) were guaranteed annually for the Member States from 2024 to 2027,^28^ leaving other pharmaceutical companies out of the agreement. This decision potentially poses a risk for the regional prevention of COVID-19, as the Europe-wide vaccination strategy at now depends on a single vaccine manufacturer.^29^

## Vaccines available for COVID-19

1. Platforms used in vaccines for COVID-19

**Fig. 2:**
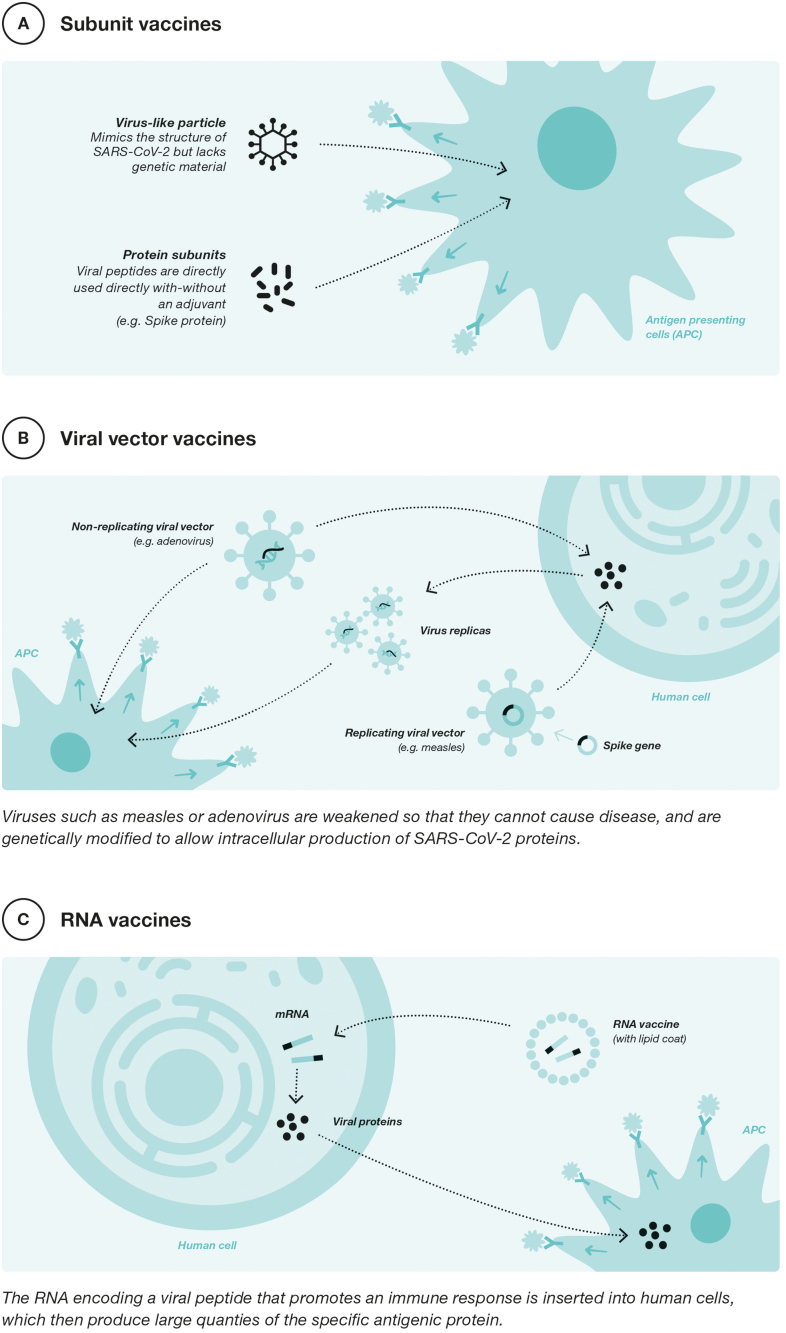
COVID-19 vaccine platforms. Adapted from P. Buchy, Y. Buisson, O. Cintra et al.

In the global response to COVID-19, vaccine development has been key to containing the pandemic. Different platforms have been used in the development of SARS-CoV-2 vaccines. The following figure shows the three most used platforms currently in use in Europe for vaccination against COVID-19 (Fig. 2).

Currently, there are 5 vaccines approved by the European Medicines Agency (EMA)^30^ (Table 2).

**Table 2:** Vaccines approved in Europe.

Platform	Vaccine	Company	Variants	Updated to EMA 2023–2024 recommendations
mRNA	Spikevax	Moderna Biotech	Original variant	×
			Original variant + BA.1 variant (updated)	×
			Original variant + BA.4-5 variants (updated)	×
			Omicron XBB.1.5 variant (updated)	✓

mRNA	Comirnaty	BioNTech	Original variant	×
			Original variant + BA.1 variant (updated)	×
			Original variant + BA.4-5 variant (updated)	×
			Omicron XBB.1.5 variant (updated)	✓

Protein subunit	Nuvaxovid	Novavax.	Original variant	×
			Omicron XBB.1.5 variant (updated)	✓

Protein subunit	Bimervax	HIPRA	Alpha and beta variants	×

Viral vector	Jcovden	Janssen-Cilag	Original variant	×

Vaccine development during the pandemic started with 2 platforms, messenger ribonucleic acid (mRNA) and viral vector vaccines. The mRNA vaccines, such as those developed by Pfizer-BioNTech (BNT162b2) and Moderna (mRNA-1273), have been the most widely used vaccines worldwide against COVID-19.^31^ These vaccines contain mRNA to produce the SARS-CoV-2 virus antigen of interest, namely the S-glycoprotein (from the SARS-CoV-2 spike protein). This glycoprotein is recognized by the immune system, which triggers a robust and specific immune response against it that neutralizes the virus.^32^ The use of these vaccines has been rarely associated with the occurrence of self-limited myocarditis/pericarditis and other more serious events requiring hospitalization.^33^

Viral vector platforms use vectors such as modified adenoviruses to carry DNA copies of SARS-CoV-2 mRNA to produce the S-glycoprotein antigen in human body cells.^32^ Examples of vaccines using this technology include ChAdOx1 nCov-19 (Ofxord-Astrazeneca, although it is no longer available in Europe and its distribution in Spain has been discontinued) or Ad26.COV2-S (Janssen, which also is not available).^32^ The use of these vaccines has been associated on rare occasions with the appearance of thrombosis and thrombocytopenia.^34^

As of December 2021, the EMA approved protein subunit vaccines for COVID-19. The first of these was developed by Novavax (Nuvaxoid) and more recently another developed by HIPRA (BIMERVAX®).^35^, ^36^ These vaccines consist of specific fragments of SARS-CoV-2 virus spike proteins, produced by recombinant technology. In the case of Nuvaxovid utilizing the Baculovirus platform in Sf9 cells of *Spodoptera frugiperda*, and in the case of BIMERVAX® using the expression of SARS-CoV2 antigens in Chinese hamster ovary-derived cell culture. Both systems faithfully reproduce the composition of the SARS-CoV-2 viral antigens and each is used together with an adjuvant that helps to enhance and broaden the immune response to the vaccine antigens. Both vaccines are adjuvanted: Nuvaxovid with MATRIX-M a novel saponin-based adjuvant derived from the bark of the *Quillaja saponaria Molina* tree and Bimervax®, (with variants B.1.351 and B.1.17), using the SQBA adjuvant, which is similar in composition to the classical MF59 adjuvant.^36^

Protein subunit platforms have several advantages in the context of COVID-19 vaccination. They are appropriate for immunocompromised patients and generate high neutralizing antibody titers compared to mRNA virus vaccines.^37^ The Novavax vaccine, in addition to demonstrating high efficacy in phase 3 trials, has a lower risk of reactogenicity side effects.^38^, ^39^, ^40^, ^41^ Protein subunit vaccines generally show favorable immunogenicity when administered as heterologous booster doses. In addition, they do not require deep-freeze storage for large-scale distribution.^37^

2. Efficacy of current vaccines against Omicron lineage variants.

Both the vaccines developed with an mRNA platform (BioNTech and Moderna) with viral vectors (Astrazeneca and Janssen) as well as Novavax's protein subunit vaccine demonstrated high efficacy and a favorable safety profile with significant variations among them, in their application for the Wuhan ancestral strain, against which these vaccines were designed.^42^ The exception is the HIPRA vaccine which does not yet have such efficacy data.

The efficacy of the first COVID-19 vaccines (BNT162b2, mRNA-1273, AZD1222 and Ad26.COV2.S) begins to decline at approximately 3 months although they continue to protect against hospitalization and death for 6–9 months in the period before the appearance of the Omicron variant.^43^, ^44^ Studies support the need for booster doses with mRNA vaccines every 6 months to maintain adequate protection against Omicron lineage variants.^45^

Other studies have observed a decrease in the efficacy of mRNA vaccines against the Omicron lineage variants of SARS-CoV-2 virus. They indicate that the neutralizing capacity of sera from individuals vaccinated with mRNA vaccines may be significantly reduced against Omicron compared to earlier variants of the virus, such as Delta.^46^ These findings highlight the importance of continuous virological surveillance and adoption of vaccination strategies to adapt to the evolution of the virus and ensure effective protection against prevalent and clinically important emerging variants.

The Novavax vaccine, a protein based vaccine, has demonstrated robust neutralizing capacity against Omicron lineage variants in animal models, including against S-glycoprotein mutations.^47^ In various clinical trials, it has demonstrated efficacy with long-lasting protection at a follow-up of 7.5 months.^39^, ^40^, ^41^ Additionally, NVX-CoV2373 shows fewer reactogenicity local and systemic adverse events than mRNA vaccines, suggesting a better tolerability profile.^48^ These findings suggest that the Novavax vaccine could maintain an effective immune response against Omicron lineage variants, making it a very useful option for reducing the severity of cases and achieving safe and effective vaccinations.

3. Adjuvants included in COVID-19 vaccines

Adjuvants play a crucial role in the efficacy of vaccines in general and against COVID-19 in particular; by enhancing, broadening, expanding and directing the body's immune response against SARS-CoV-2. These compounds, incorporated into vaccine formulations, act as enhancers of the immune response (homo- and heterotypic), increasing the duration and magnitude of the protective response against specific antigens of the virus.^49^

3.1 Importance of adjuvants

The ability to enhance the individual immune response, in addition to improving the logistical capabilities of vaccine use, is essential for the use of adjuvants in future vaccines.

The inclusion of adjuvants in COVID-19 vaccines is relevant for the following reasons:

***Enhancing vaccine response in the population***. Adjuvants play an important role in the efficacy of COVID-19 vaccines by enhancing the immune response in both the population of healthy adults and young people and in at-risk groups including older adults, or those with underlying diseases.^50^
***Reduction in the number of doses***. Adjuvants contribute to the reduction of the number of doses required to achieve an optimal immune response, which improves the efficacy and speed of immunization and simplifies the logistics of vaccination.^49^
***Stimulation of the immune system***. Adjuvants stimulate the cellular immune response in addition to the humoral response, activating adjuvant and effector T cells, which contributes to a more complete protection against viral infection and its variants.^51^

3.2 Types of adjuvants

The two most commonly used adjuvants in current COVID-19 vaccines are MATRIX-M and SQBA. The latter adjuvant acts predominantly on CD4+ Th2-dominant T cells,^52^ has been used in influenza vaccines and has been investigated for application in COVID-19 vaccines. It is similar in composition to MF59, which is a squalene-oil emulsion, and is included in the HIPRA vaccine.^36^

The Matrix-M adjuvant, included in the Novavax vaccine, is based on plant-derived saponins derived from the bark of the *Q. saponaria Molina* tree. This adjuvant stimulates both humoral and cellular responses and is Th1 dominant,^53^ enhancing the cellular efficacy of the vaccine. It has been used and has shown good results in vaccines such as malaria and influenza.^54^

## Future recommendations for post-pandemic vaccination seasons

**Fig. 3:**
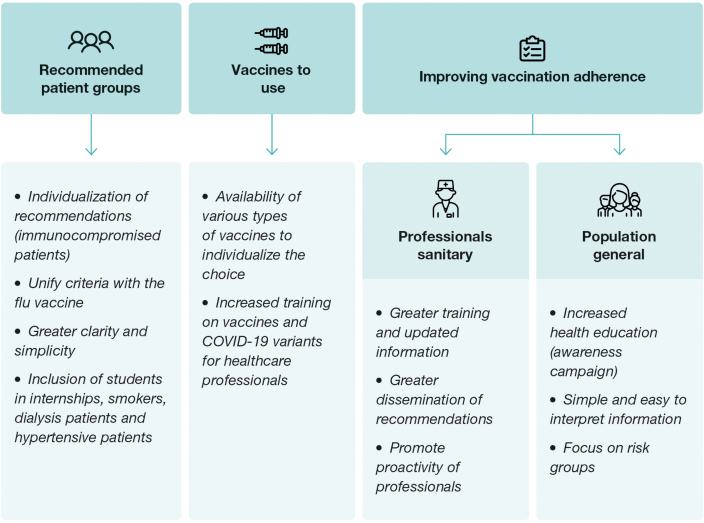
Recommendations to implement in COVID-19 vaccination for next vaccination seasons.

In the context of future vaccination against COVID-19 in Spain, it is important to strategically address several considerations to improve vaccination rates (Fig. 3) and to allow the use of different vaccines. To this end, current vaccination recommendations should be adopted with the aim of overcoming the challenges and barriers that are contributing to the decline in vaccination coverage. It is necessary, in this context, to present the advantages and disadvantages of current vaccine and vaccination options.

Given the continuous evolution of the virus and the appearance of new variants, it is necessary to consider flexibility in the selection of vaccines, prioritizing those that demonstrate efficacy against emerging variants and that allow for a robust and long-lasting immune response in the population. This comprehensive and adaptive approach is essential to strengthen the national response to the pandemic and mitigate its impact on public health.^25^

### Vaccination recommendations

For future vaccination campaigns, a series of recommendations should be implemented with the aim of improving the effectiveness and accessibility of vaccination against COVID-19. First, the importance of further individualization of recommendations should be emphasized, especially for immunocompromised patients, who may require specific vaccination strategies to optimize their immune response and protection against the virus.^55^ In this sense, it is also recommended to maintain the booster vaccination age to ensure enhanced protection in this more vulnerable segment of the population. In addition, the need for greater clarity and simplicity in the recommendations should be emphasized, including decision algorithms to help health professionals in the selection and administration of vaccines in the future.

To this end, it is essential to unify the recommendations for vaccination against COVID-19 with those currently in place for influenza vaccination, which would facilitate the implementation of coordinated strategies and the maximization of vaccination coverage in the population. In this line of argument, the inclusion of additional groups in the vaccination recommendations, such as medical students,^20^ smokers, dialysis patients and patients with hypertension is proposed.^24^

To ensure adequate implementation of the recommendations, it is necessary to provide more training to health professionals on risk groups and recommended vaccination strategies. The lack of sufficient and adequate information on the COVID-19 vaccine during the last year showed the need to improve communication and dissemination of accurate, up-to-date, and timely information on the available vaccines, their characteristics, and their safety.^56^

Finally, the need to use updated vaccines that can cope with emerging variants of the virus must be emphasized. In addition the importance of having access to different types of vaccines in order to be able to individualize the therapeutic decision, which is now limited by the restrictions imposed by the APA.^25^, ^57^ It is also important to highlight the need to clarify concepts and doubts about COVID-19 vaccines in the new post-pandemic period to promote greater acceptance and confidence in the population.^58^ These recommendations, taken together, seek to strengthen the COVID-19 vaccination campaign and maximize its impact on public health protection.

In the transition to the post-pandemic phase, vaccines are needed to address the specific challenges of this new endemic scenario. An ideal vaccine for this stage should be long-lasting, provide sustained protection against the virus, have minimal reactogenicity, and adapt effectively to emerging variants of the virus. In addition, it is essential that these vaccines be aligned with international recommendations established by reputable regulatory agencies such as the European Medicines Agency (EMA) and the World Health Organization (WHO); who have already recommended adapting vaccines to target the JN.1 family of Omicron subvariants because of the increased circulation of this variant for the 2024–2025 season.^16^, ^17^ Following these guidelines ensures not only the safety and efficacy of vaccines, but also greater public confidence and better protection for the population against future epidemiological challenges. An international consensus on updated vaccines could encourage the use of updated vaccines in a uniform manner across countries.

### Strategies to improve adherence to vaccination for healthcare professionals and the general population

Health education about COVID-19 and vaccination has been a constant concern among healthcare professionals and the general population.^59^ Health professionals have faced limited dissemination of official information about COVID-19 disease, including vaccine coverage and poorly accessible information about circulating variants and updated vaccines. In addition, low dissemination at certain levels of information on vaccine efficacy and tolerance, together with poor training and lack of knowledge of recommendations, has contributed to a low vaccination rate among health professionals,^60^ negatively impacting patient confidence in the vaccination process.

In the general population, in addition to deficiencies in health education, there is a lack of accessible, simple and easy-to-interpret information. The improvement of the epidemiological situation and the presence of less virulent predominant variants have led to a situation of lower awareness of individual risk and the spread of different types of misinformation.^58^

To address these deficiencies, various communication and information strategies are proposed. This includes the implementation of simple and regular communication, improving awareness campaigns and focusing on the at-risk population. The use of attractive dissemination tools and the promotion of the concept of seasonal vaccination against COVID-19 are suggested. It is crucial to provide up-to-date information on vaccine efficacy and safety, with easy-to-interpret examples, as well as to empower all health professionals to be active agents in promoting vaccination.

It is important that vaccination campaigns are led jointly by governmental institutions, scientific societies and patient associations, in order to provide a unified and coherent message. Collaboration among these entities will allow greater effectiveness in disseminating reliable information and promoting vaccination as an essential measure to prevent and reduce the burden of disease and its consequences.

## Conclusions

A comprehensive approach to COVID-19 vaccination in Spain for future seasons requires a series of strategic and adaptive measures. Recognizing the shortcomings in some of the current strategies, it is essential to improve the individualization of recommendations, unify vaccination guidelines against COVID-19 and influenza, and promote greater training and communication among health professionals and the general population.

In addition, given the constant evolution of the virus and the appearance of new variants, it is essential to consider flexibility in the selection of vaccines, prioritizing those that demonstrate greater efficacy/effectiveness against the predominant circulating strains and that allow a robust and long-lasting immune response.

The availability of updated vaccines with the dominant variants and the inclusion of adjuvanted vaccines are key elements to improve their efficacy and acceptance, thus contributing to routine immunization against COVID-19 in future years, mitigating its impact on public health.

## Funding

This article has been funded by Novavax. The authors have not received any financial contribution for their participation in the article.

## Conflicts of interest

PA has received honoraria for advisory boards from Novavax. MFP has received honoraria for advisory boards from HIPRA and Novavax, consulting fees from AstraZeneca and for lectures and educational events from MSD and GSK. AGM has received honoraria for advisory boards and lectures from Pfizer, GSK, Novavax, Sanofi, MSD, HIPRA and Seqirus. JGR has received honoraria for advisory boards, consulting fees and lectures from Pfizer, Moderna, MSD, Novavax, Seqirus, AstraZeneca and GSK. CHG has received honoraria for advisory boards and lectures from GSK, HIPRA, Pfizer, Moderna, Sanofi and Novavax and for educational events from Pfizer, Sanofi and Seqirus. RM has received honoraria for advisory boards, consulting fees and lectures from Pfizer, GSK, Novavax, Sanofi and MSD. AT has received honoraria for advisory boards from Pfizer and Sanofi, for scientific committees from Novavax and for lectures from Moderna and Sanofi. ROL has received honoraria for consulting fees and scientific committees from Moderna, MSD, Pfizer and Novavax.
